# The global burden and trends of maternal sepsis and other maternal infections in 204 countries and territories from 1990 to 2019

**DOI:** 10.1186/s12879-021-06779-0

**Published:** 2021-10-18

**Authors:** Liyuan Chen, Qi Wang, Yun Gao, Jinxiang Zhang, Sheng Cheng, Huilong Chen, Zhilin Zeng, Zhongxian Wang

**Affiliations:** 1 Department of Obstetrics and Gynecology, Wuhan No.1 Hospital, Wuhan, China; 2 Department of Respiratory and Critical Care Medicine, Tongji Hospital, Tongji Medical College, Huazhong University of Science and Technology, Wuhan, China; 3 Ezhou Centers for Disease Control and Prevention, Ezhou, China; 4 Department of Spine Surgery, The Second Hospital affiliated to Guangdong Medical University, Guangdong Medical University, Zhanjiang, China; 5 Department of Respiratory Diseases, University-Town Hospital of Chongqing Medical University, Chongqing, China; 6 Department and Institute of Infectious Diseases, Tongji Hospital, Tongji Medical College, Huazhong University of Science and Technology, Wuhan, China

**Keywords:** Maternal sepsis and other maternal infections, Global burden disease, Incidence, Death, Disability-adjusted life year, Maternal mortality ratio

## Abstract

**Background:**

Maternal sepsis and other maternal infections (MSMI) have considerable impacts on women’s and neonatal health, but data on the global burden and trends of MSMI are limited. Comprehensive knowledge of the burden and trend patterns of MSMI is important to allocate resources, facilitate the establishment of tailored prevention strategies and implement effective clinical treatment measures.

**Methods:**

Based on data from the Global Burden of Disease database, we analysed the global burden of MSMI by the incidence, death, disability-adjusted life year (DALY) and maternal mortality ratio (MMR) in the last 30 years. Then, the trends of MSMI were assessed by the estimated annual percentage change (EAPC) of MMR as well as the age-standardized rate (ASR) of incidence, death and DALY. Moreover, we determined the effect of sociodemographic index (SDI) on MSMI epidemiological parameters.

**Results:**

Although incident cases almost stabilized from 1990 to 2015, the ASR of incidence, death, DALY and MMR steadily decreased globally from 1990 to 2019. The burden of MSMI was the highest in the low SDI region with the fastest downward trends. MSMI is still one of the most important causes of maternal death in the developed world. Substantial diversity of disease burden and trends occurred in different regions and individual countries, most of which had reduced burden and downward trends. The MMR and ASR were negatively correlated with corresponding SDI value in 2019 in 204 countries/territories and 21 regions.

**Conclusion:**

These findings highlight significant improvement in MSMI care in the past three decades, particularly in the low and low-middle SDI regions. However, the increased burden and upward trends of MSMI in a few countries and regions are raising concern, which poses a serious challenge to maternal health. More tailored prevention measures and additional resources for maternal health are urgently needed to resolve this problem.

**Supplementary Information:**

The online version contains supplementary material available at 10.1186/s12879-021-06779-0.

## Background

Pregnant and recently pregnant women easily develop infections and are particularly prone to rapid progression to sepsis [1, 2]. Maternal sepsis is a life-threatening organ dysfunction caused by a dysregulated host response to infections during pregnancy, childbirth, post-abortion or the postpartum period [3]. Infection, especially sepsis contributes significantly to global morbidity and mortality, particularly in vulnerable populations [4]. Maternal infections can have serious considerable impacts on the health of women and neonates. For women, maternal infections can cause chronic pelvic inflammatory disease, ectopic pregnancy, infertility and death [5]. Annually, one million neonatal deaths are attributed to maternal infections or sepsis [4, 6, 7]. Infections or sepsis are preventable causes of maternal morbidity and mortality. However, the WHO Global Maternal Sepsis Study Research Group reported that approximately 70 pregnant or recently pregnant women per 1000 live births needed hospital management due to maternal infections, and that infection-related maternal deaths contributed to more than half of the intrahospital deaths in 2017 [8]. Furthermore, maternal sepsis accounted for approximately 11% of maternal deaths globally between 2003 and 2009 [9]. Last, 2019 GBD data estimate almost 21 million incident cases and 17 thousand deaths from maternal sepsis and other maternal infections (MSMI) worldwide [10]. In addition, the incidence and death of maternal sepsis showed geographic distribution differences around the world [10, 11]. One study delineated that 23% of all maternal deaths were sepsis-related in the United States, which meant that developed countries were not immune to this serious problem as well [12].

As one of the major public health issues worldwide, MSMI imposes appreciable socioeconomic burdens on individuals and societies. To achieve the sustainable development goals and move towards ending preventable maternal mortality, a comprehensive understanding of the burden and trends of MSMI, stratified by age, sociodemographic index (SDI), regions and countries, is a valuable reference for public health leaders, researchers, funding agencies and clinical doctors. In this study, we presented the statistical data of MSMI globally, in 21 regions and in 204 countries/territories from 1999 to 2019.

## Methods

### Overview

The Global Burden of Disease (GBD) database is a comprehensive project that measures epidemiological levels and trends among communicable diseases, noncommunicable diseases, and injuries across the world. In the GBD 2019, 369 diseases and injuries were studied in 204 countries and territories and 21 regions from 1990 to 2019 [10]. The general methodology for estimating the burden of diseases for GBD 2019 have been described in detail in previous studies [10, 13, 14]. Detailed descriptions of the methods are presented in the Additional file 1.

### Definition

Maternal sepsis and other maternal infections (MSMI) are including two part. Maternal sepsis is defined as a temperature < 36 °C or > 38 °C and clinical signs of shock including systolic blood pressure < 90 mmHg and tachycardia > 120 bpm. Other maternal infections are defined as any maternal infections excluding HIV, sexually transmitted infections, or are not believed to have epidemiologic relationship with pregnancy. Examples include urinary tract infections, mastitis, candidiasis, and bacterial vaginosis during pregnancy [10].

The disability-adjusted life year (DALY), equal to the sum of the years lived with disability and the years of life lost, is a summary measure of population health that accounts for both mortality and nonfatal health consequences. DALY was designed as the unit of analysis for measuring the relative magnitude of losses of healthy life associated with specific causes [15]. DALY have been proposed by the World Bank and the WHO as a measure of the global impact of disease on individual illness status [16].

The sociodemographic index (SDI) is a composite indicator of income per capita, years of schooling, and fertility rate in females younger than 25 years. The SDI is ranging from 0 to 1. The larger the SDI, the more developed the country [17, 18]. The SDI of different countries and territories are presented in the Additional file 2.

Age-standardized rate (ASR) refers to the method of statistical processing of demographic data according to the same standard age composition [17]. The purpose is to eliminate the influence of different age composition of the population and ensure the comparability of statistical indicators [19].

Maternal mortality ratio (MMR) is the number of maternal deaths per 100,000 livebirths [20].

### Data source

The incidence, death as well as DALY, and corresponding ASR in 204 countries/territories from 1990 to 2019 were downloaded from the GBD database.

Information on age, SDI and geographic location were also collected to further analyze the burden of MSMI. Women are divided into nine groups according to age, including the 15 year, 15–19 year, 20–24 year, 25–29 year, 30–34 year, 35–39 year, 40–44 year, 45–49 year and above 49 year. Then, countries and territories are divided into five regions according to the SDI, including low, low-middle, middle, high-middle and high. Moreover, the world was divided into 21 regions according to the geographic location.

The Institutional Review Board of Wuhan No.1 Hospital, Tongji Medical College, Huazhong University of Science & Technology determined that approval was waived because of publicly available data. GBD 2019 complies with the Guidelines for Accurate and Transparent Health Estimates Reporting statement [21].

### Statistical analysis

Annual number of incident cases, death, DALY, and corresponding ASR (number per 100,000 population) as well as MMR were used to describe the MSMI burden.

ASR were calculated on the basis of the following formula: $ASR=\frac{{\sum }_{\mathrm{i}=1}^{A}{a}_{i}{w}_{i}}{{\sum }_{\mathrm{i}=1}^{A}{w}_{i}}$ × 100,000. The ASR (per 100,000 population) is equal to the sum of the product of the specific age ratio (a_i_) in age group i and the number (or weight) (w_i_) of the selected reference standard population group i divided by the sum of number (or weight) of the standard population.

The estimated annual percentage change (EAPC) was calculated using the following regression model to assess the trends in ASR: Y = α + βX + ε, where Y refers to ln(ASR), X represents calendar year, ε means error term, and β determines the positive or negative trends in ASR. The EAPC could be given by 100*(exp(β) − 1) [22]. The ASR was considered to be on the rise when the estimation of EAPC and its lower boundary of 95% uncertainty interval [UI] were both positive. On the contrary, the ASR was considered to be in a downward trend when EAPC and its upper boundary of 95% UI were both negative. Otherwise, the ASR was considered to be stable over time.

The Spearman's correlation coefficients were used to assess the relationships between the ASR/EAPC and SDI. In the correlation analysis, if the Pearson correlation coefficient was < 0 and the P value was < 0.05, there was a significant negative correlation between the two variables. The P-value less than 0.05 is considered statistical significance.

## Results

### The incidence of MSMI

**Fig. 1:**
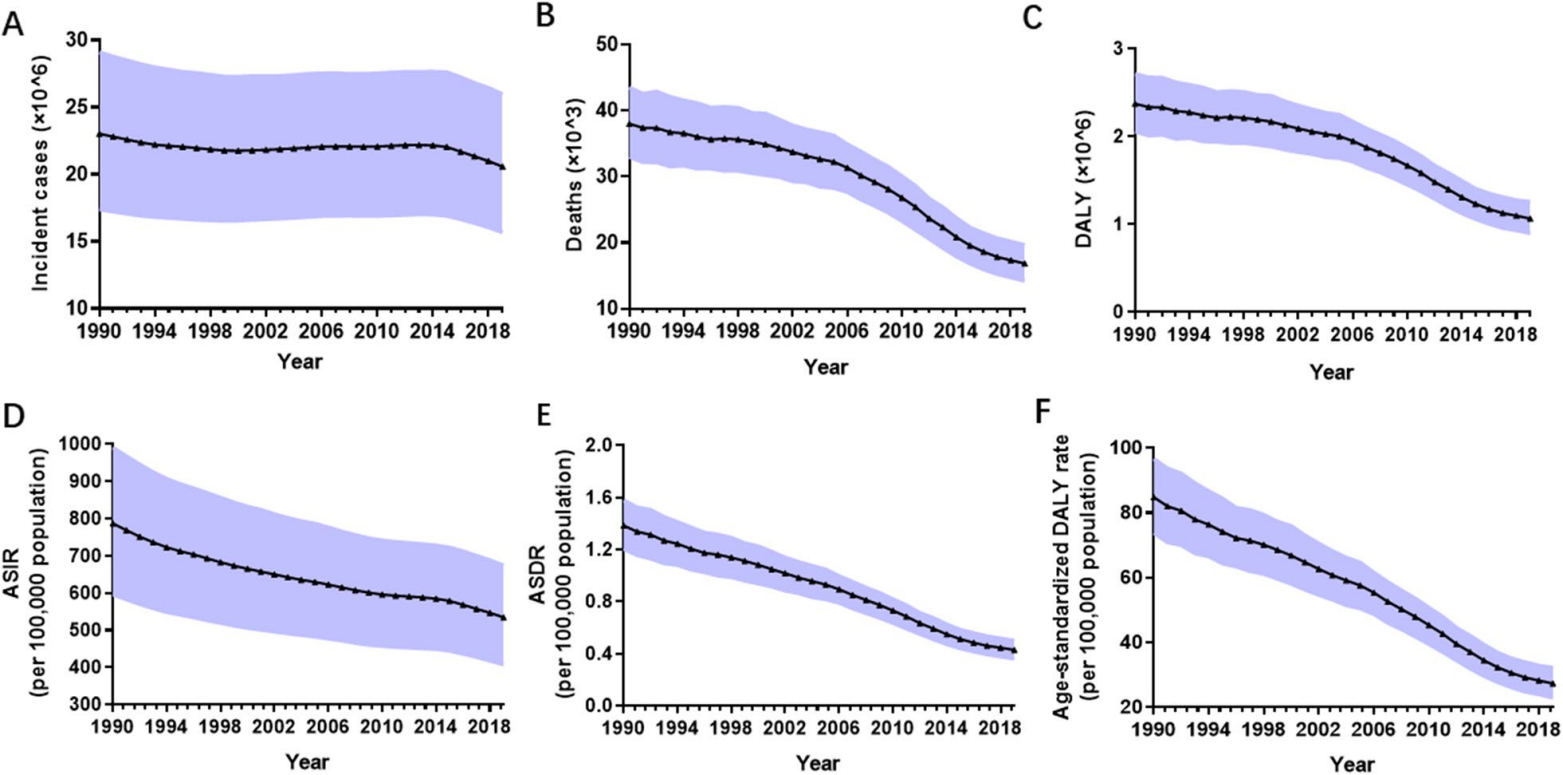
The burden and trends of MSMI globally from 1990 to 2019. **A** The change of incident cases. **B** The change of death number. **C** The change of DALY. **D** The change of ASIR per 100,000 population. **E** The change of ASDR per 100,000 population. **F** The change of age-standardized DALY rate per 100,000 population. The black triangle represent value in corresponding year and shading shows 95% uncertainty intervals. Note: *MSMI* maternal sepsis and other maternal infections; *DALY* disability-adjusted life-years; *ASIR* age-standardized incident rate; *ASDR* age-standardized death rate

**Fig. 2:**
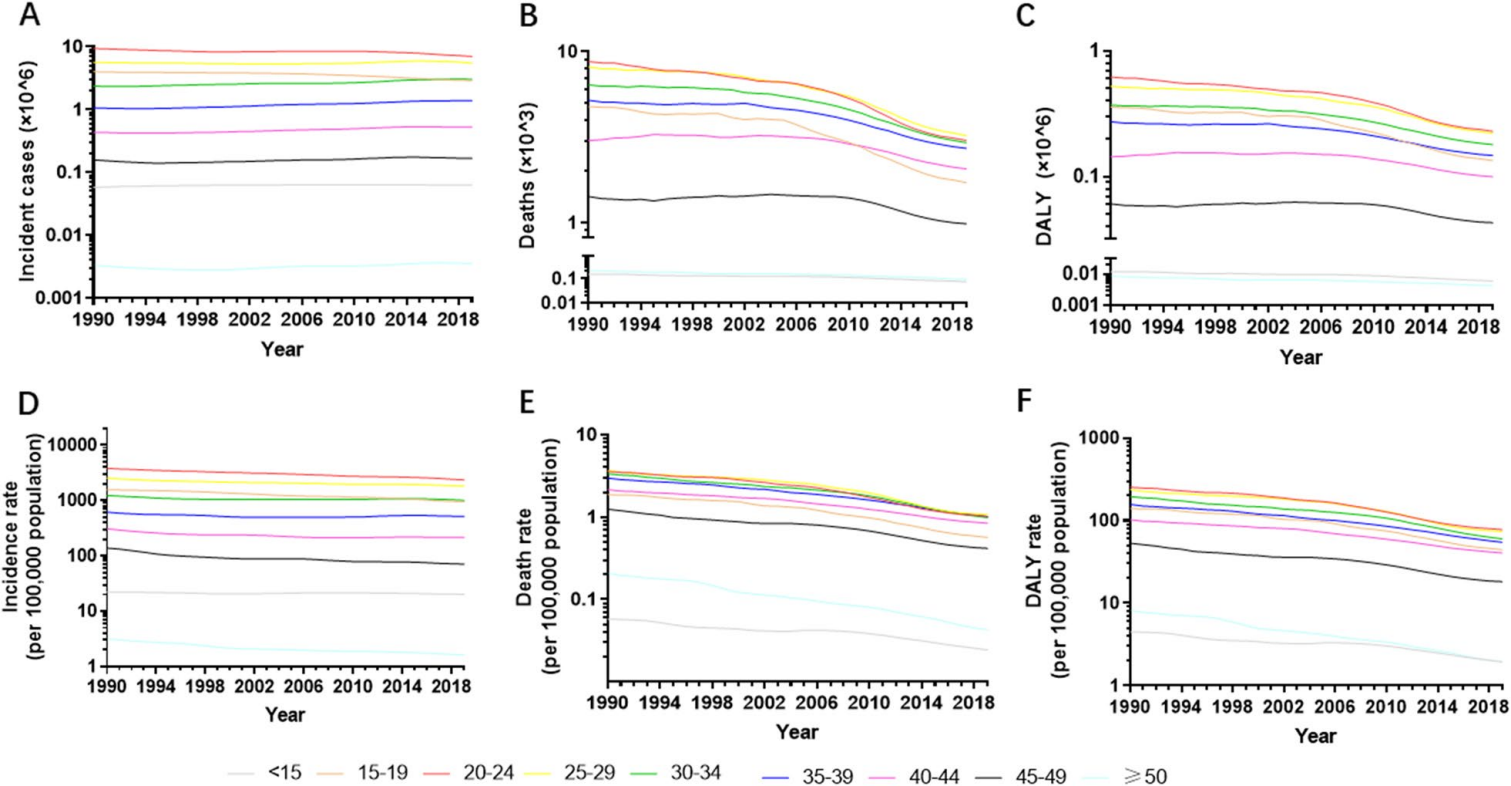
The burden and trends of MSMI worldwide in different ages in the past 30 years **A** The change of incident cases. **B** The change of death number. **C** The change of DALY. **D** The change of ASIR per 100,000 population. **E** The change of ASDR per 100,000 population. **F** The change of age-standardized DALY rate per 100,000 population. Note: *MSMI* maternal sepsis and other maternal infections; *DALY* disability-adjusted life-years; *ASIR* age-standardized incident rate; *ASDR* age-standardized death rate

Globally, there were 23,029,127 incident cases of MSMI (95% UI 17,399,801–29,084,559) in 1990 and 20,569,889 incident cases (95% UI 15,688,621–25,972,495) in 2019 (Fig. 1A and Additional file 3: Table S1). The global incident cases remained almost constant from 1990 to 2015 and subsequently decreased from 2016 to 2019 (Fig. 1A and Additional file 3: Table S1). In the globe, the ASIR of MSMI declined by 38.48% gradually from 787 in 1990 to 534 in 2019 with an EAPC value of − 1.17 (95% UI − 1.23 to − 1.11) (Fig. 1D and Additional file 4: Table S2). The largest incident cases and ASIR were observed in the 20–24 year group, followed by 25–29 year group (Fig. 2A, D and Additional file 5: Table S3). There was little annual variability in incident cases or ASIR.

**Fig. 3:**
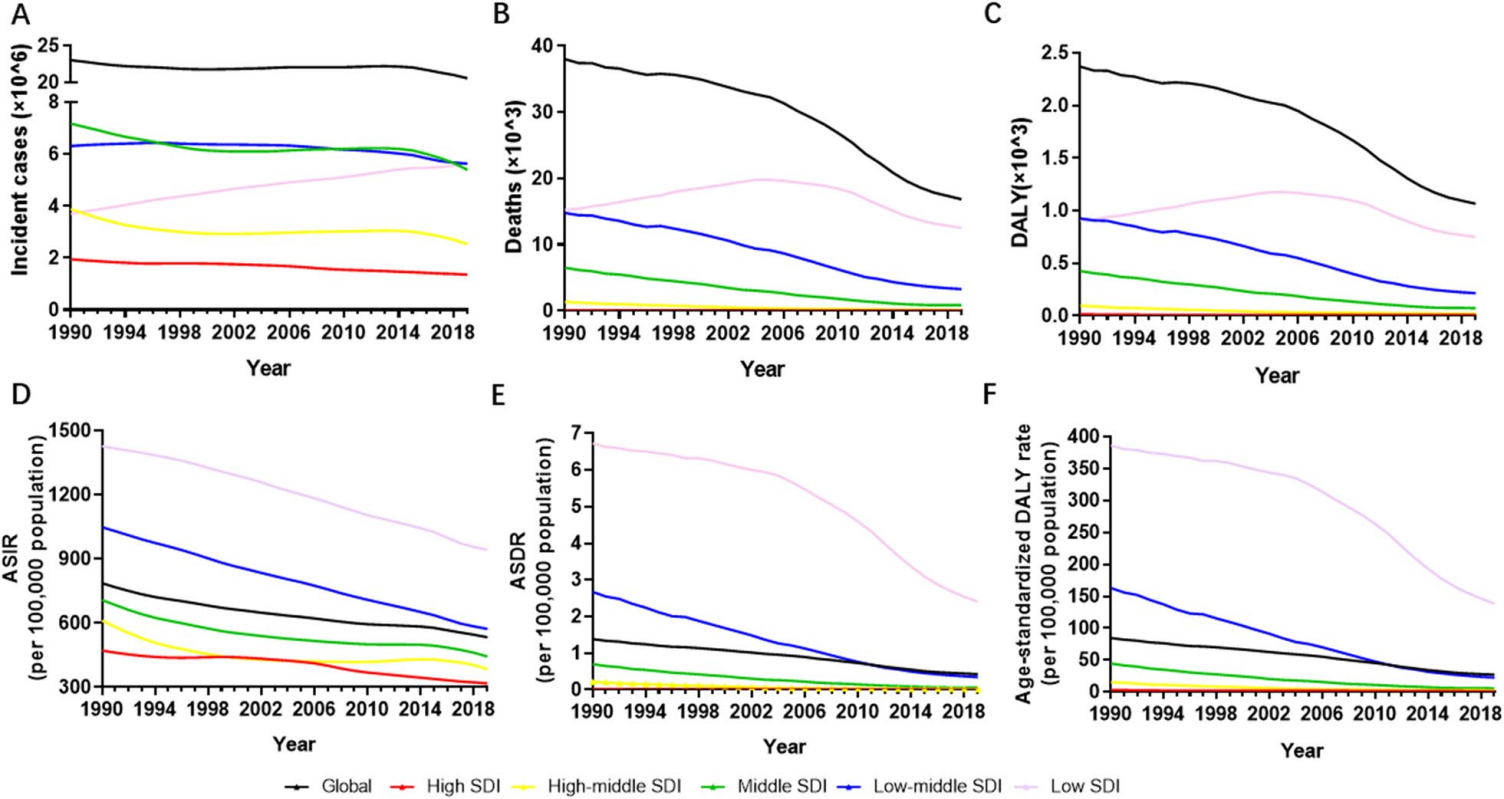
The burden and trends of MSMI in five SDI quintiles globally from 1990 to 2019. **A** The change of incident cases. **B** The change of death number. **C** The change of DALY. **D** The change of ASIR. **E** The change of ASDR. **F** The change of age-standardized DALY rate. Note: *MSMI* maternal sepsis and other maternal infections; *SDI* sociodemographic index; *ASIR* age-standardized incident rate per 100,000 population; *ASDR* age-standardized death rate per 100,000 population; *DALY* disability-adjusted life-years per 100,000 population

At the regional level, the middle SDI and low-middle SDI regions had the largest number of incident cases of MSMI until 2017. The low SDI region suffered from a rapid increase in incident cases of MSMI. From 2018, the low SDI and low-middle SDI regions upgraded to the top two in terms of burden of incident cases (Fig. 3A and Additional file 6: Table S4). Once the impacts of ageing were removed by converting counts to age-standardized rates, a steady decrease in ASIR was noted in all SDI regions. The low SDI and low-middle SDI regions had the highest ASIR and the most rapid decline in the past 30 years (Fig. 3D and Additional file 6: Table S4).

Subgroup analysis by geographical zone indicated that South Asia suffered from the most incident cases (6,219,971 in 1990 and 5,495,269 in 2017) (Additional file 7: Table S5). As shown in Table 1, Central Sub-Saharan Africa and Eastern Sub-Saharan Africa had the highest ASIR, whereas only Australasia and Tropical Latin America were on the rise of ASIR (EAPC of Australasia: 0.63, 95% UI 0.43–0.83; EAPC of Tropical Latin America: 0.62, 95% UI 0.35–0.89), the others were in a downward trend. The ASIR decreased the most in South Asia (EAPC − 2.45, 95% UI − 2.53 to − 2.37) and in North Africa and the Middle East (EAPC − 2.04, 95% UI − 2.11 to − 1.97) (Table 1).

**Table 1:** The value and temporal trends of ASIR and ASDR in different region in 1990 and 2019

Characteristics	ASIR (95% UI) per 100,000 population			ASDR (95% UI) per 100,000 population		
	1990	2019	EAPC	1990	2019	EAPC
Overall	787.04 (595.71 to 990.76)	534.21 (407.34 to 673.73)	− 1.17 (− 1.23 to − 1.11)	1.38 (1.20 to 1.58)	0.43 (0.36 to 0.50)	− 4.00 (− 4.33 to − 3.67)
East Asia	513.12 (365.88 to 685.77)	246.48 (178.28 to 324.49)	− 1.56 (− 1.99 to − 1.12)	0.27 (0.21 to 0.33)	0.008 (0.006 to 0.011)	− 11.78 (− 12.19 to − 11.36)
Central Asia	1058.34 (800.56 to 1287.07)	780.96 (587.98 to 962.35)	− 0.75 (− 1.06 to − 0.44)	0.18 (0.16 to 0.21)	0.058 (0.048 to 0.071)	− 3.96 (− 4.42 to − 3.50)
South Asia	1093.12 (819.21 to 1390.73)	544.50 (403.6 to 704.75)	− 2.45 (− 2.53 to − 2.37)	3.13 (2.54 to 3.83)	0.29 (0.22 to 0.37)	− 8.39 (− 9.03 to − 7.74)
Southeast Asia	754.40 (564.50 to 981.72)	489.28 (363.22 to 636.03)	− 1.46 (− 1.51 to − 1.40)	0.92 (0.76 to 1.15)	0.11 (0.09 to 0.14)	− 7.16 (− 7.54 to − 6.78)
High-income Asia Pacific	198.19 (145.80 to 262.86)	129.56 (103.94 to 158.00)	− 1.91 (− 2.12 to − 1.71)	0.05 (0.04 to 0.06)	0.001 (0.001 to 0.001)	− 12.05 (− 13.68 to − 10.39)
Oceania	949.10 (709.71 to 1212.15)	834.08 (621.23 to 1082.10)	− 0.45 (− 0.47 to − 0.43)	3.03 (2.20 to 3.98)	1.44 (1.03 to 1.96)	− 1.98 (− 2.44 to − 1.52)
Australasia	405.26 (293.39 to 502.26)	417.76 (335.62 to 520.30)	0.63 (0.43 to 0.83)	0.01 (0 to 0.01)	0.002 (0.001 to 0.002)	− 4.91 (− 5.78 to − 4.02)
Eastern Europe	775.75 (592.49 to 946.67)	657.30 (499.90 to 820.73)	0.40 (− 0.14 to 0.94)	0.06 (0.05 to 0.07)	0.010 (0.007 to 0.012)	− 6.86 (− 7.32 to − 6.41)
Central Europe	661.47 (540.53 to 772.68)	376.47 (317.09 to 444.32)	− 1.47 (− 1.77 to − 1.18)	0.05 (0.05 to 0.06)	0.003 (0.002 to 0.003)	− 11.16 (− 11.78 to − 10.54)
Western Europe	393.06 (300.67 to 488.33)	335.87 (259.23 to 422.40)	− 0.16 (− 0.28 to − 0.04)	0.02 (0.01 to 0.02)	0.001 (0.001 to 0.002)	− 7.45 (− 8.29 to − 6.59)
Central Latin America	885.25 (664.91 to 1134.45)	606.92 (461.28 to 773.30)	− 1.11 (− 1.34 to − 0.88)	0.65 (0.59 to 0.71)	0.11 (0.09 to 0.14)	− 6.09 (− 6.58 to − 5.61)
Southern Latin America	967.01 (759.98 to 1196.39)	680.98 (540.78 to 833.05)	− 1.07 (− 1.18 to − 0.96)	0.52 (0.44 to 0.60)	0.13 (0.11 to 0.16)	− 4.19 (− 4.66 to − 3.72)
High-income North America	645.20 (519.36 to 769.68)	375.65 (303.02 to 446.13)	− 2.10 (− 2.47 to − 1.73)	0.011 (0.009 to 0.013)	0.009 (0.007 to 0.011)	0.49 (− 0.05 to 1.03)
Andean Latin America	1042.60 (790.53 to 1315.38)	659.08 (494.53 to 834.52)	− 1.62 (− 1.69 to − 1.55)	2.03 (1.71 to 2.41)	0.25 (0.17 to 0.33)	− 7.62 (− 8.08 to − 7.17)
Tropical Latin America	593.35 (460.32 to 747.08)	581.88 (463.53 to 702.13)	0.62 (0.35 to 0.89)	0.59 (0.49 to 0.69)	0.13 (0.11 to 0.16)	− 3.97 (− 4.49 to − 3.43)
Caribbean	771.43 (577.11 to 983.67)	577.84 (431.79 to 734.99)	− 0.97 (− 1.02 to − 0.91)	1.36 (1.00 to 1.90)	1.49 (1.01 to 2.07)	1.24 (0.87 to 1.61)
North Africa and Middle East	1163.07 (898.90 to 1426.43)	612.29 (467.29 to 765.32)	− 2.04 (− 2.11 to − 1.97)	1.79 (1.52 to 2.11)	0.32 (0.23 to 0.42)	− 6.24 (− 6.50 to − 5.98)
Central sub-Saharan Africa	1541.23 (1190.64 to 1942.23)	973.77 (749.33 to 1245.80)	− 1.42 (− 1.57 to − 1.27)	6.11 (4.47 to 8.02)	5.48 (4.05 to 7.29)	0.98 (− 0.12 to 2.09)
Eastern sub-Saharan Africa	1490.86 (1138.75 to 1872.87)	954.81 (727.63 to 1214.78)	− 1.53 (− 1.59 to − 1.47)	8.53 (6.77 to 10.83)	2.31 (1.87 to 2.84)	− 4.65 (− 5.13 to − 4.16)
Southern sub-Saharan Africa	841.79 (651.00 to 1057.97)	612.10 (463.80 to 783.59)	− 0.91 (− 0.99 to − 0.82)	1.60 (1.33 to 1.88)	0.74 (0.54 to 1.00)	− 0.52 (− 1.84 to − 0.81)
Western sub-Saharan Africa	1185.92 (935.25 to 1465.29)	842.43 (661.68 to 1043.01)	− 1.25 (− 1.36 to − 1.14)	4.45 (3.63 to 5.31)	1.70 (1.33 to 2.15)	− 3.52 (− 3.85 to − 3.18)

*ASIR* age-standardized incident rate per 100,000 population; *ASDR* age-standardized death rate per 100,000 population; *EAPC* annual percentage change

**Fig. 4:**
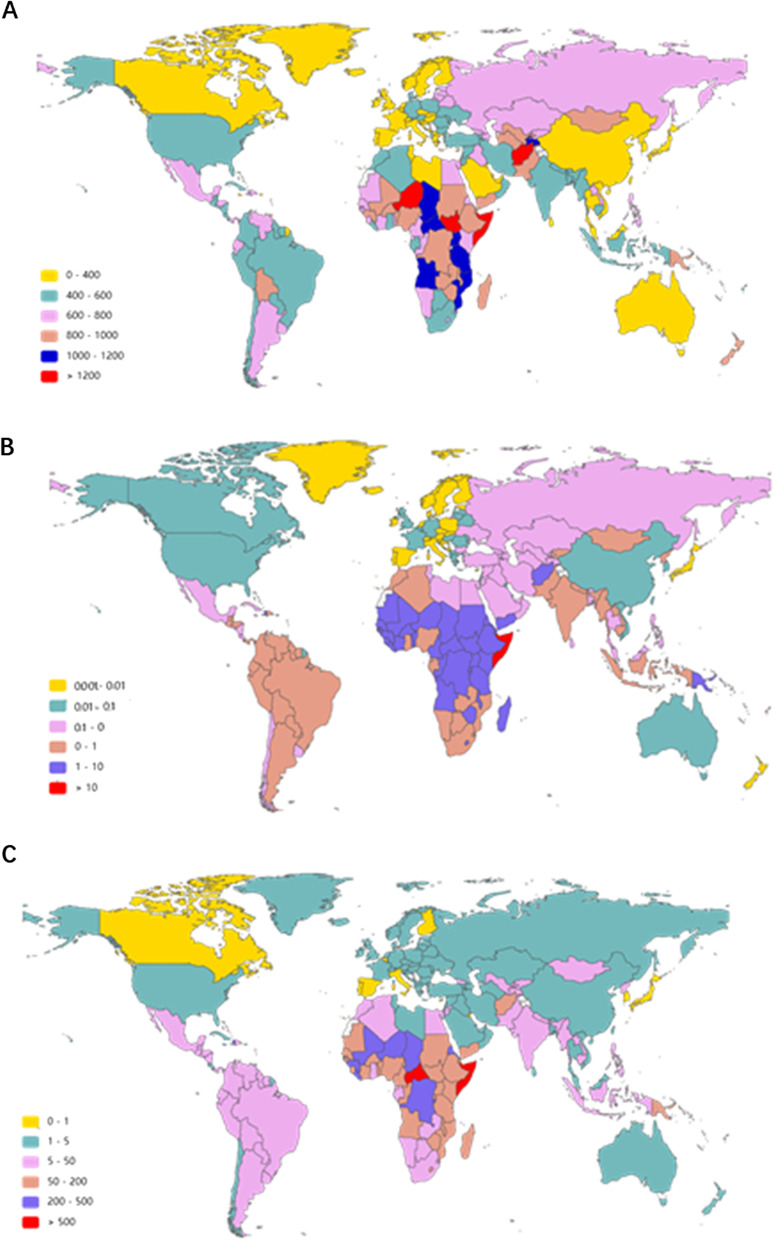
The age-standardized rate of MSMI in 204 countries and territories in 2019. **A** The ASIR of MSMI globally in 2019. **B** The ASDR of MSMI globally in 2019. **C** The age-standardized DALY rate of MSMI globally in 2019. Note: *MSMI* maternal sepsis and other maternal infections; *ASR* age-standardized rate; *ASIR* age-standardized incident rate per 100,000 population; *ASDR* age-standardized death rate per 100,000 population; *DALY* disability-adjusted life-years per 100,000 population

**Fig. 5:**
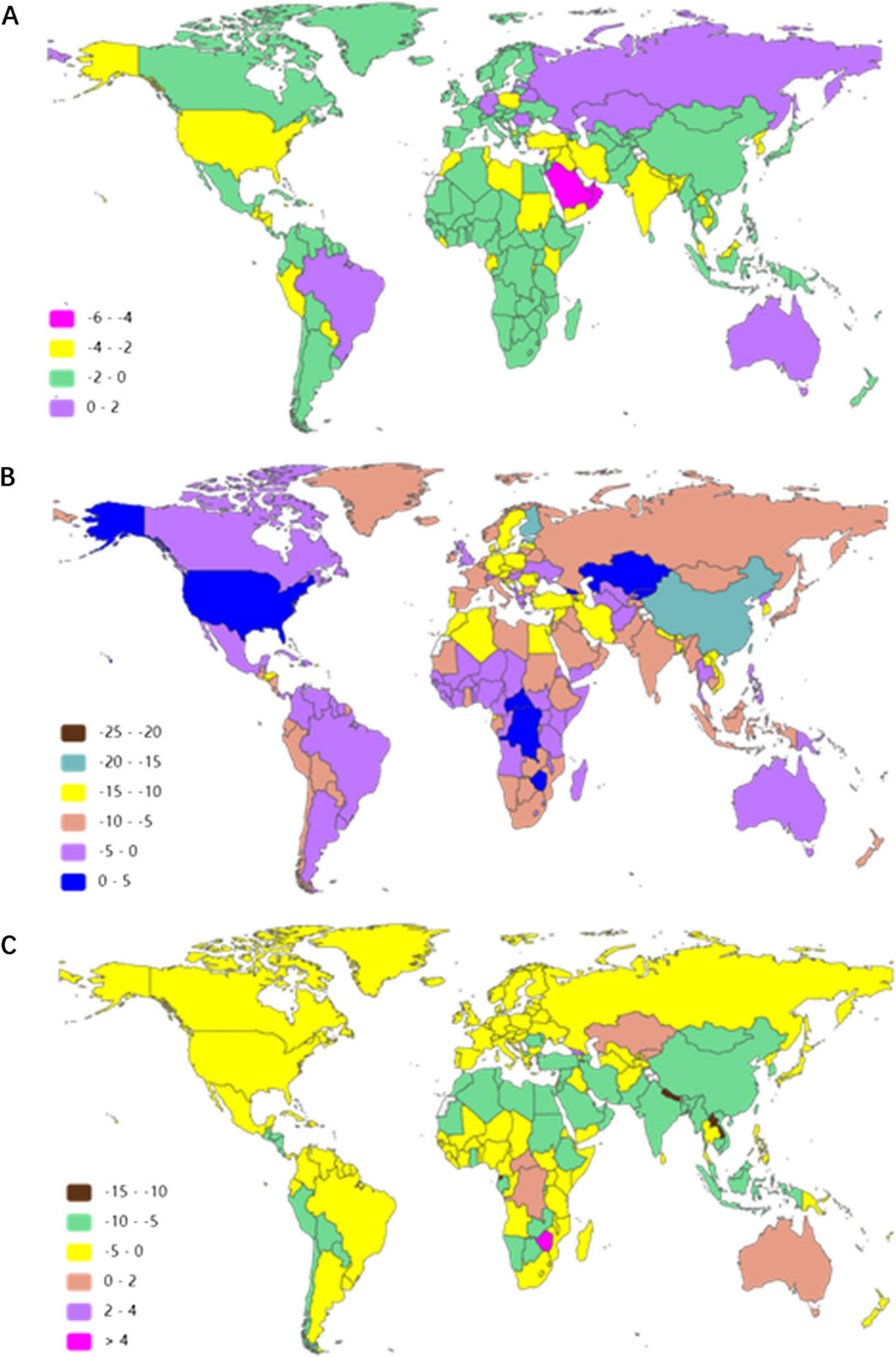
The EAPC of MSMI in 204 countries and territories in the last three decades. **A** The EAPC of ASIR per 100,000 population in the last three decades. **B** The EAPC of ASDR per 100,000 population. **C** The EAPC of age-standardized DALY rate per 100,000 population. Note: *MSMI* maternal sepsis and other maternal infections; *EAPC* annual percentage change; *ASIR* age-standardized incident rate per 100,000 population; *ASDR* age-standardized death rate per 100,000 population; *DALY* disability-adjusted life-years per 100,000 population

At the country/territory level, Somalia, Niger, South Sudan and Afghanistan presented the highest MSMI ASIR in 2019 (1328, 1296, 1249 and 1240, respectively) (Fig. 4A and Additional file 8: Table S6). Australia, Georgia, the Russian Federation and Romania were in the top 4 in the upward trend of ASIR in the past 30 years with EAPC values of 1.37 (95% UI 1.01–1.74), 0.91 (95% UI 0.38–1.43), 0.71 (95% UI 0.15–1.27) and 0.48 (95% UI 0.09–0.88), respectively (Fig. 5A and Additional file 9: Table S7).

### MSMI death

At a global level, the number of deaths due to MSMI showed a downward trend in the last 30 years from 38,027 (95% UI 33,014–43,506) cases in 1990 to 16,840 (95% UI 14,224–19,633) cases in 2019 (Fig. 1B and Additional file 3: Table S1). The same change applies to women of different ages in death number and ASDR, with the greatest number and rate in the 20–24 year group, followed by the 25–29 year group (Fig. 2B, E and Additional file 5: Table S3). The ASDR of MSMI was reduced by 68.96% gradually from 1.38 per 100,000 population in 1990 to 0.43 per 100,000 population in 2019. (Fig. 1E, Table 1 and Additional file 4: Table S2).

As reported in Fig. 3B, the low SDI region had the heaviest burden in terms of the number of deaths in the past three decades. Furthermore, the number of deaths in the low SDI region increased from 1990 to 2004 and subsequently declined after 2005 (Fig. 3B and Additional file 6: Table S4). All SDI regions exhibited a downward trend in ASDR, especially in the low SDI region with a sharp decrease (Fig. 3E and Additional file 6: Table S4).

The region with the largest number of deaths changed from South Asia in 1990 (16,848 cases, 95% UI 13,528–20,629) to Eastern Sub-Saharan Africa in 2019 (4396 cases, 95% UI 3,584–5,378) (Additional file 7: Table S5). As shown in Table 1, the highest ASDR occurred in Eastern Sub-Saharan Africa (8.53, 95% UI 6.77–10.83) in 1990, and in Central Sub-Saharan Africa (5.48, 95% UI 4.05–7.29) in 2019. All regions (except for the Caribbean, high-income North America and Central Sub-Saharan Africa) demonstrated downward trends in the three decades. However, the Caribbean region displayed an upward trend with an EAPC value of 1.24 (95% UI 0.87–1.61) (Table 1). At the various country/territory levels, the ASDR was highest in Somalia (11.82, 95% UI 7.70–17.02) and lowest in Iceland (7.52 × 10^–5^, 95% UI 5.62 × 10^–5^–9.88 × 10^–5^) in 2019 (Fig. 4B and Additional file 8: Table S6). The ASDR was elevated only in Georgia, Kazakhstan, Kyrgyzstan, the Democratic Republic of the Congo, Zimbabwe and United States and declined in others in the past 30 years. In addition, the fastest growth of ASDR was in Zimbabwe (EAPC 4.10, 95% UI 2.40–5.83) and Georgia (EAPC 3.57, 95% UI 1.60–5.59), whereas the fastest decrease was in Bosnia and Herzegovina (EAPC − 22.24, 95% UI − 23.99 to − 20.46) and Finland (EAPC − 16.44, 95% UI − 18.02 to − 14.83) (Fig. 5B and Additional file 9: Table S7).

### MSMI DALY

There were 2,370,496 DALY numbers (95% UI 2,053,615–2,710,598) due to MSMI in 1990 globally and 1,064,670 DALY numbers (95% UI 895,636–1,256,185) in 2019 (Fig. 1C and Additional file 3: Table S1). From 1990 to 2019, the DALY number decreased gradually by 55.09% at all ages. The age-standardized DALY rate decreased sharply by 67.77% around the world in the past three decades (Fig. 1F and Additional file 4: Table S2). Similarly, the decreasing trend held true for diverse age groups in the absolute number and ASR of DALY (Fig. 2C, F and Additional file 5: Table S3).

Except for the low SDI region, the DALY number of the MSMI decreased gradually in different SDI regions, with the highest decrease by 82.97% in the middle SDI region and the lowest decrease by 52.10% in high SDI region (Fig. 3C and Additional file 6: Table S4). The DALY number increased in the low SDI region from 1990 to 2005 and began to fall thereafter in 2006. The age-standardized DALY rate varies considerably across the world, with the highest rate observed in the low SDI region (386.55 in 1990 and 138.84 in 2019) and with the lowest rate in the high SDI region (3.39 in 1990 and 1.58 in 2019) (Fig. 3F and Additional file 6: Table S4).

In terms of geographical region, the highest DALY number occurred in South Asia in 1990 (1,066,308, 95% UI 855,921–1,302,359), but in Eastern Sub-Saharan Africa in 2019 (261,751, 95% UI 214,313–318,317) (Additional file 7: Table S5). For the age-standardized DALY rate of MSMI, the region with the highest value changed from Eastern Sub-Saharan Africa in 1990 to Central Sub-Saharan Africa 2019 (Table 2). In addition, the age-standardized DALY rate of MSMI significantly declined in most regions, with the largest decrease in East Asia (EAPC − 8.16, 95% UI − 8.77 to − 7.55). In 21 regions, only the Caribbean was elevated during the study period, with an EAPC value of 1.16 (95% UI 0.79–1.53) (Table 2).

**Table 2:** The value and temporal trends of age standard DALY rate and MMR in different region in 1990 and 2019

Characteristics	Age-standardized DALY rate (95% UI)			MMR (95% UI) per 100,000 livebirths		
	1990	2019	EAPC	1990	2019	EAPC
Overall	84.83 (73.76 to 96.71)	27.34 (22.97 to 32.30)	− 3.89 (− 4.20 to − 3.57)	27.53 (23.90 to 31.49)	12.44 (10.51 to 14.51)	− 0.57 (− 0.64 to − 0.50)
East Asia	18.08 (14.40 to 22.64)	1.50 (0.90 to 2.42)	− 8.16 (− 8.77 to − 7.55)	7.43 (5.84 to 9.29)	0.38 (0.27 to 0.51)	− 0.23 (− 0.25 to − 0.21)
Central Asia	14.85 (11.99 to 18.70)	6.56 (4.66 to 9.21)	− 2.82 (− 3.04 to − 2.60)	3.25 (2.78 to 3.74)	1.56 (1.29 to 1.90)	− 0.09 (− 0.11 to − 0.06)
South Asia	194.32 (157.31 to 236.81)	19.51 (15.14 to 24.55)	− 8.13 (− 8.73 to − 7.52)	47.03 (37.76 to 57.58)	8.37 (6.45 to 10.74)	− 0.54 (− 0.56 to − 0.51)
Southeast Asia	56.85 (47.06 to 71.44)	8.65 (6.94 to 10.91)	− 6.49 (− 6.80 to − 6.19)	17.98 (14.82 to 22.62)	3.76 (3.03 to 4.58)	− 1.51 (− 1.58 to − 1.44)
High-income Asia Pacific	3.49 (2.79 to 4.37)	0.59 (0.31 to 1.03)	− 5.42 (− 6.41 to − 4.42)	2.11 (1.77 to 2.52)	0.06 (0.05 to 0.07)	− 0.47 (− 0.62 to − 0.31)
Oceania	178.00 (128.58 to 233.14)	86.19 (62.41 to 115.75)	− 1.92 (− 2.37 to − 1.47)	41.64 (29.80 to 54.89)	23.35 (16.84 to 31.59)	− 0.45 (− 0.60 to − 0.30)
Australasia	1.99 (1.07 to 3.36)	1.76 (0.84 to 3.20)	0.001 (− 0.22 to 0.22)	0.22 (0.17 to 0.27)	0.06 (0.05 to 0.08)	− 0.06 (− 0.07 to − 0.05)
Eastern Europe	6.57 (4.74 to 9.36)	3.24 (1.78 to 5.52)	− 2.32 (− 2.61 to − 2.04)	2.13 (1.79 to 2.51)	0.39 (0.30 to 0.51)	− 0.10 (− 0.11 to − 0.08)
Central Europe	5.93 (4.40 to 8.04)	1.70 (0.89 to 2.91)	− 4.34 (− 4.86 to − 3.82)	1.97 (1.70 to 2.29)	0.13 (0.11 to 0.17)	− 0.07 (− 0.08 to − 0.06)
Western Europe	2.61 (1.75 to 3.95)	1.44 (0.72 to 2.59)	− 1.32 (− 1.65 to − 1.00)	0.74 (0.65 to 0.85)	0.06 (0.06 to 0.07)	− 0.02 (− 0.02 to − 0.01)
Central Latin America	41.80 (37.68 to 46.34)	9.10 (6.96 to 11.74)	− 5.24 (− 5.71 to − 4.78)	11.24 (10.28 to 12.29)	3.42 (2.65 to 4.42)	− 0.26 (− 0.30 to − 0.22)
Southern Latin America	33.90 (28.77 to 40.22)	10.28 (7.99 to 13.05)	− 3.58 (− 3.99 to − 3.17)	12.60 (10.71 to 14.51)	4.64 (3.83 to 5.58)	− 0.23 (− 0.27 to − 0.18)
High-income North America	3.25 (1.89 to 5.42)	2.05 (1.24 to 3.32)	− 1.54 (− 1.89 to − 1.20)	0.36 (0.29 to 0.43)	0.37 (0.29 to 0.46)	0.04 (0.02 to 0.05)
Andean Latin America	120.34 (101.68 to 142.69)	16.77 (12.25 to 22.04)	− 7.16 (− 7.60 to − 6.71)	30.89 (26.09 to 36.87)	6.20 (4.38 to 8.28)	− 0.99 (− 1.13 to − 0.85)
Tropical Latin America	36.31 (30.76 to 42.43)	10.57 (8.55 to 13.04)	− 3.19 (− 3.69 to − 2.69)	13.28 (11.14 to 15.73)	4.98 (4.16 to 5.97)	− 0.20 (− 0.25 to − 0.15)
Caribbean	81.31 (60.17 to 110.66)	87.23 (59.70 to 120.41)	1.16 (0.79 to 1.53)	27.66 (20.39 to 38.01)	44.12 (29.94 to 61.68)	0.87 (0.73 to 1.01)
North Africa and Middle East	106.56 (90.91 to 125.34)	20.81 (15.63 to 26.97)	− 5.90 (− 6.12 to − 5.68)	23.11 (19.67 to 27.32)	8.39 (6.16 to 11.06)	− 0.58 (− 0.61 to − 0.55)
Central sub-Saharan Africa	346.47 (257.10 to 450.00)	305.01 (227.71 to 397.23)	0.90 (− 0.19 to 2.00)	52.80 (38.89 to 68.84)	71.54 (53.63 to 92.72)	1.78 (0.96 to 2.60)
Eastern sub-Saharan Africa	473.54 (377.85 to 598.18)	132.18 (108.08 to 161.14)	− 4.53 (− 5.01 to − 4.05)	73.57 (58.46 to 93.30)	31.04 (25.30 to 37.97)	− 1.64 (− 1.80 to − 1.48)
Southern sub-Saharan Africa	95.31 (80.20 to 111.18)	45.95 (33.89 to 60.92)	− 0.49 (− 1.76 to 0.79)	27.82 (23.22 to 32.81)	19.40 (14.14 to 26.01)	0.13 (− 0.16 to 0.42)
Western sub-Saharan Africa	254.10 (207.46 to 302.21)	99.96 (79.33 to 126.60)	− 3.42 (− 3.75 to − 3.08)	39.88 (32.47 to 47.72)	22.39 (17.59 to 28.45)	− 0.73 (− 0.82 to − 0.64)

*DALY* disability-adjusted life-years; *MMR* number of maternal deaths per 100,000 livebirths; *EAPC* annual percentage change

In terms of countries, the age-standardized DALY rate was highest in Somalia (656.45, 95% UI 433.86–934.27), followed by the Central African Republic (542.47, 95% UI 362.86–796.05) in 2019 (Fig. 4C and Additional file 8: Table S6). As presented in Fig. 5C and Additional file 9: Table S7, Zimbabwe, Georgia, the Democratic Republic of the Congo, Kazakhstan and Australia had positive EAPC values in 2019. In addition, the age-standardized DALY rate exhibited downward trends in most countries/territories, with the fastest decrease in Equatorial Guinea (EAPC − 12.56, 95% UI − 11.83 to − 13.29).

### MSMI MMR

**Fig. 6:**
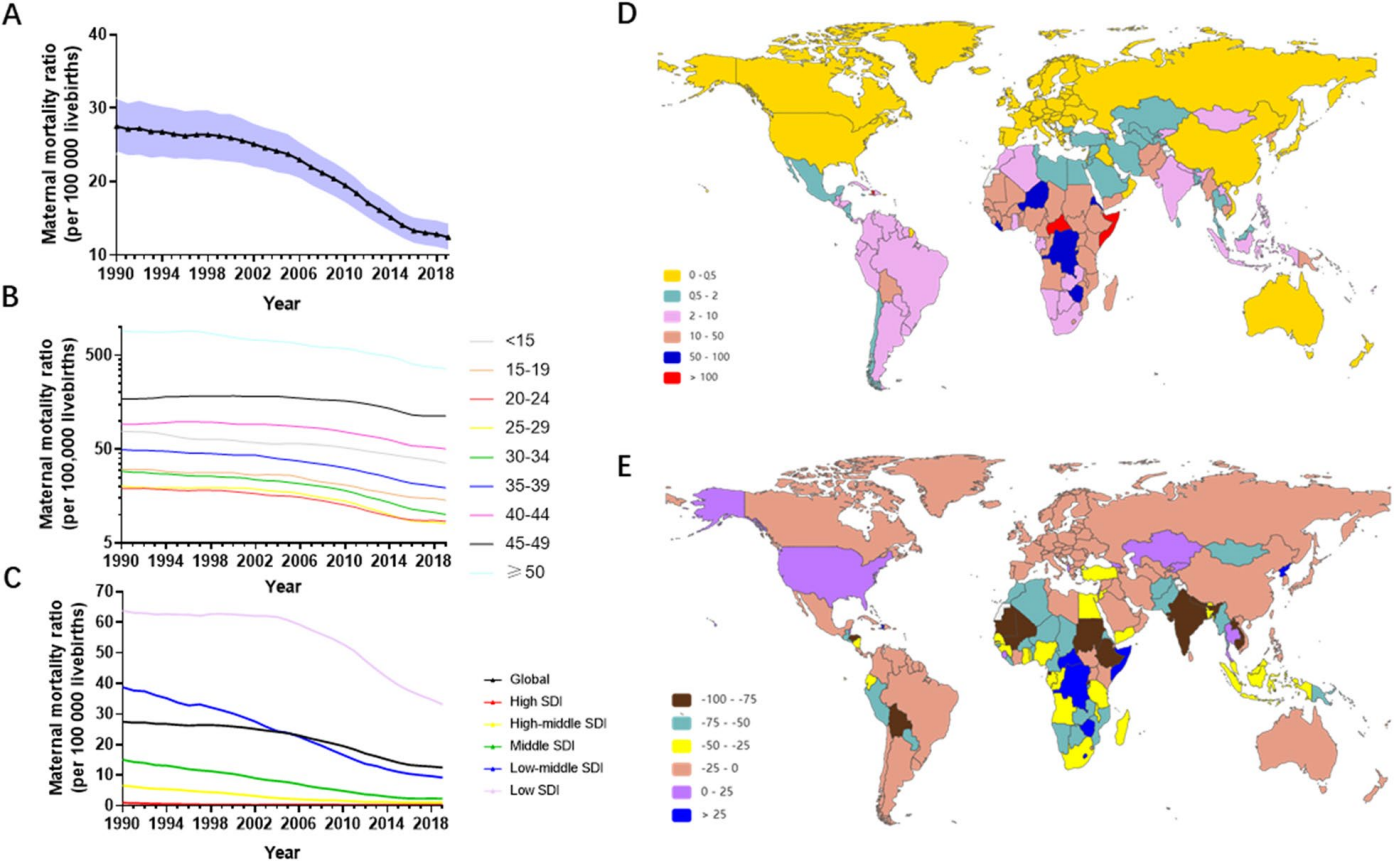
The MMR of MSMI across the world in the last 30 years. **A** The change of MMR in all age from 1990 to 2019. **B** The change of MMR in different age groups. **C** The change of MMR in five SDI regions in 2019. **D** The MMR of MSMI globally per 100,000 livebirths in 2019. **E** The EAPC of MMR. The black triangle represent value in corresponding year and shading shows 95% uncertainty intervals. Note: *MSMI* maternal sepsis and other maternal infections; *MMR* number of maternal deaths per 100,000 livebirths; *EAPC* annual percentage change

Globally, the MMR of MSMI decreased 54.80%, from 27.53 (95% UI 23.90 to − 31.49) in 1990 to 12.44 (95% UI 10.51–14.51) in 2019 (Fig. 6A and Additional file 10: Table S8). The largest MMR was observed in the above 49 year group, followed by the 45–49 and 40–44 year groups around the world (Fig. 6B and Additional file 10: Table S8). There was little annual decline in the MMR of the different groups, except for the 45–49 and 40–44 year groups.

Subgroup analysis by sociodemographic factors demonstrated that the low SDI, low-middle SDI and middle SDI regions were the top three regions in the MMR, accounting for more than 80% (Fig. 6C and Additional file 10: Table S8).

In the subgroup analysis by geographical zone, Eastern Sub-Saharan Africa (73.57, 95% UI 58.46–93.30) and Central Sub-Saharan Africa (71.54, 95% UI 53.63–92.72) had the highest MMR in 1990 and 2019, respectively (Table 2). However, the region with the lowest burden changed from Australasia (0.22, 95% UI 0.17–0.27) in 1990 to high-income Asia Pacific (0.22, 95% UI 0.17–0.27) in 2019 (Table 2). The largest increase in MMR was observed in Central Sub-Saharan Africa (EAPC 1.78, 95% UI 0.96–2.60), followed by the Caribbean and high-income North America (Table 2).

At the country/territory level, the highest level of MMR was demonstrated in the Central African Republic (122.36, 95% UI 81.78–179.27), followed by Somalia (114.28, 95% UI 75.89–163.72), and Haiti (100.21, 95% UI 66.27–141.19) in 2019 (Fig. 6D and Additional file 10: Table S8). The annual change in MMR varied considerably across the world, with the highest elevation in the Democratic Republic of the Congo (EAPC 1913.66, 95% UI 513.33–6511.08), followed by Zimbabwe (EAPC 519.24, 95% UI 261.73–960.08) and the Central African Republic (EAPC 186.23, 95% UI 144.28–235.39) (Fig. 6E and Additional file 9: Table S7).

### The correlation between SDI and parameters assessing disease burden and trends

**Fig. 7:**
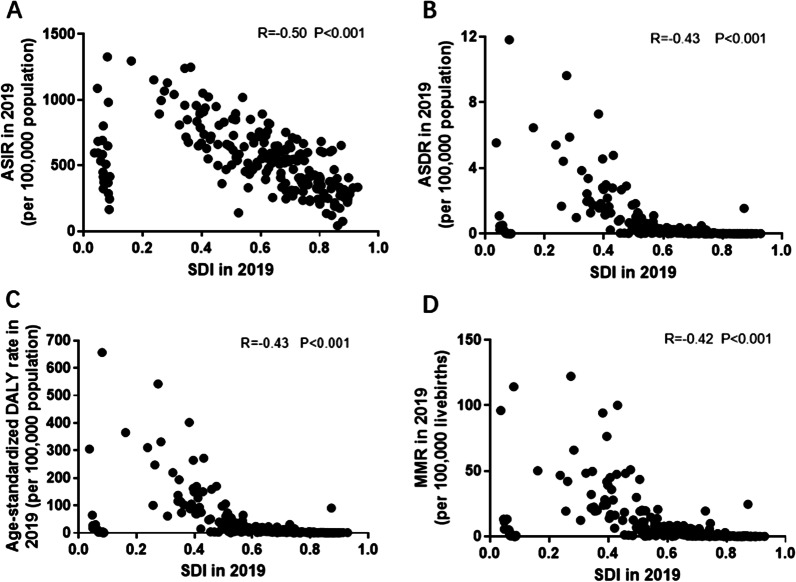
The correlation between the SDI and both ASR and MMR of MSMI in 2019. The SDI negatively correlated to the ASIR (**A**), ASDR (**B**), age-standardized DALY rate (**C**) and MMR (**D**) in 2019. Note: *MSMI* maternal sepsis and other maternal infections; *MMR* number of maternal deaths per 100,000 livebirths; *ASIR* age-standardized incident rate per 100,000 population; *ASDR* age-standardized death rate per 100,000 population; *DALY* disability-adjusted life-years per 100,000 population

For various countries/territories level, the correlation between SDI value in 2019 with ASR and MMR were examined. We observed that SDI in 2019 was negatively correlated with ASIR (R = − 0.50, P < 0.001), ASMR (R = − 0.43, P < 0.001), age-standardized DALY rate (R = − 0.43, P < 0.001) and MMR (R = − 0.42, P < 0.001) (Fig. 7A–D).

**Fig. 8:**
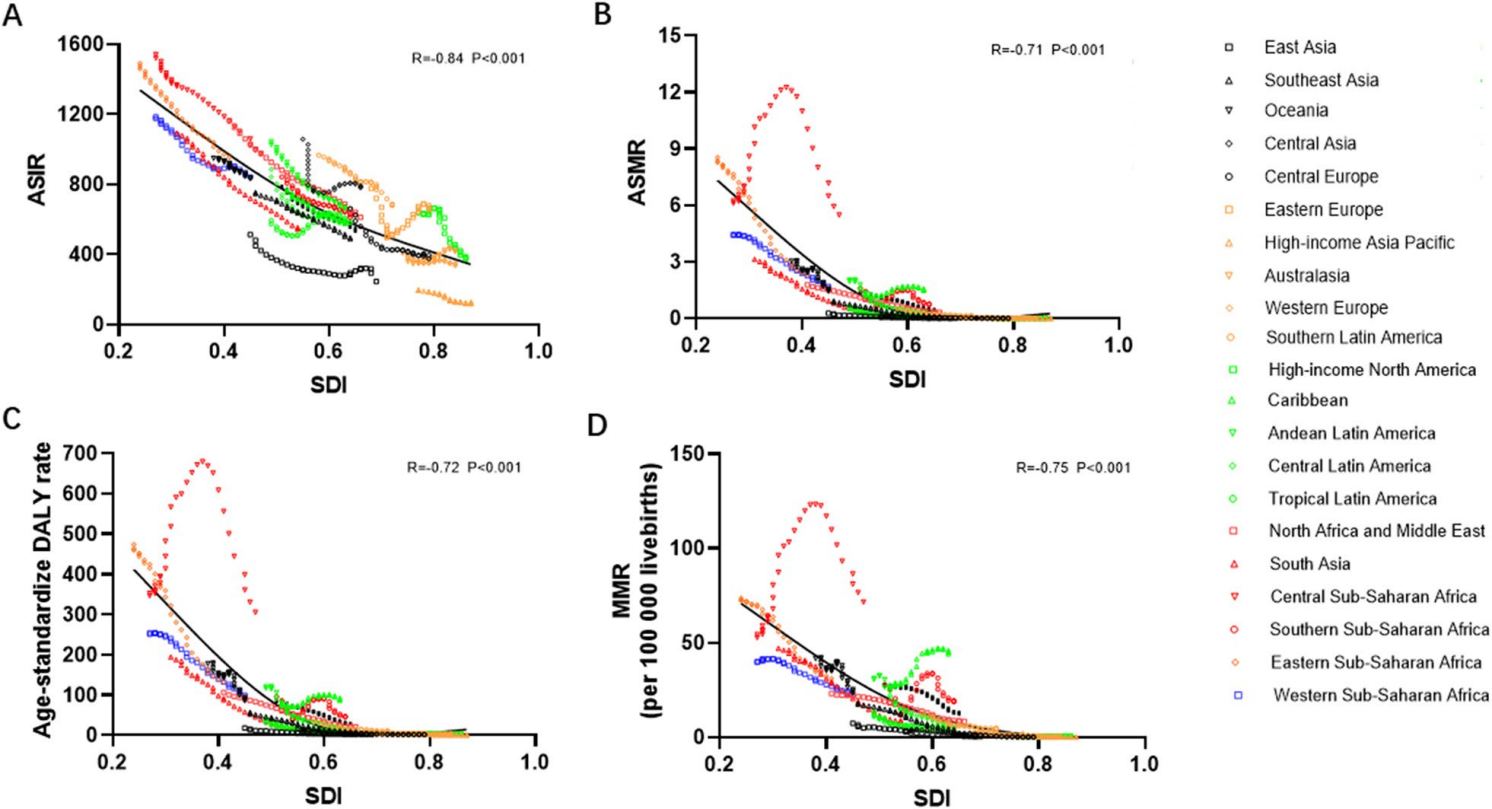
The correlation analyses between SDI and ASR or MMR of MSMI in 21 regions from 1990 to 2019. The SDI negatively correlated with the ASIR (**A**), ASDR (**B**), age-standardized DALY rate (**C**) and MMR (**D**) in 21 regions from 1990 to 2019. Note: *MSMI* maternal sepsis and other maternal infections; *ASIR* age-standardized incident rate per 100,000 population; *ASDR* age-standardized death rate per 100,000 population; *DALY* disability-adjusted life-years per 100,000 population; *MMR* number of maternal deaths per 100,000 livebirths

Similarly, we determined the correlation coefficient between ASIR in 1990 and the corresponding EAPC value. The EAPC of ASIR (R = − 0.19, P < 0.01), ASMR (R = − 0.19, P < 0.01) and the age-standardized DALY rate (R = − 0.26, P < 0.001) were negatively correlated with ASIR in 1990 (Additional file 11: Fig. S1A–C). In addition, we assessed the correlation between SDI in 2019 and EAPC values of ASR and MMR. The SDI in 2019 was negatively correlated with ASMR (R = − 0.16, P = 0.02) but did not correlate with ASIR, age-standardized DALY or MMR (Additional file 11: Fig. S1E–H). Furthermore, the correlation between SDI with ASR/MMR was investigated in 21 regions in the past three decades. The results showed that all ASR and MMR values were markedly negatively correlated with the corresponding SDI (Fig. 8A–D).

## Discussion

This study was a systematic overview of the burden and trends of MSMI at the global, regional and national levels based on the GBD 2019. The global burden of MSMI has steadily improved over the past three decades, as measured by the number or ASR of incidence, death and DALY and by the EAPC values of ASR and MMR. The number of incident cases, deaths and DALY decreased slightly or obviously in MSMI. After accounting for population growth and ageing, the ASIR, ASDR and age-standardized DALY showed steady downward trends over time. Similar change was found in MMR. For the SDI level, the burden of MSMI was the highest in the low and low-middle SDI regions with the fastest downward trend, which was several times higher than that in the high SDI region. Moreover, the burden and trends of MSMI were heterogeneous across 21 regions and 204 countries/territories, most of which had reduced disease burden. In addition, MMR and ASR were negatively correlated with corresponding SDI value in 2019 in 204 countries/territories and 21 regions. The EAPC value of ASDR was associated with both ASIR in 1990 and SDI in 2017.

In pregnancy and the puerperium, maternal physiological and immunological adaptations, designed to facilitate development of the foetus, may impair the maternal capacity to respond to infection [1, 2]. When maternal infection is out of control, it easily progresses to sepsis [23]. Sepsis is defined as a dysregulated host response to infection leading to organ dysfunction. Sepsis can involve any system in the body and is associated with irreversible multiorgan failure and ultimately death in severe forms [24]. The pathogenesis is highly complex and incompletely understood. A bedside tool for the prognosis and diagnosis of sepsis was validated in the general population, and awareness of sepsis was increased by several campaigns [25, 26]. However, pregnancy-specific management is based on those developed for the nonpregnant population, and the diagnosis of sepsis is still difficult due to physiological changes in pregnancy [4, 27, 28]. Given these challenges, improvements in sepsis-related mortality in the general population may not be observed among pregnant women. Our knowledge of the underlying pathophysiology is incomplete, which is a significant contributing factor in our inability to deal effectively with this disease.

Our study found that 182 countries and territories had negative EAPC values of MMR. Ambitious calls for a decrease in maternal mortality in the next two decades and reductions in MMRs to less than 30 in all countries have been practicable [29]. Prioritizing a reduction in maternal mortality is one of the seventeen Sustainable Development Goals (SDGs) [30]. Despite great progress in maternal health, our data suggested that at least nine countries too far to achieve the SDGs target with MMR less than 70 per 100,000 live births. Without a doubt, there was broad concern that little or no progress was being made in some areas. Our data demonstrated that 14 countries/territories still showed positive EAPC values of MMR due to MSMI until 2019. Those countries/territories may present the greatest challenge to the achievement of low SDGs. Thus, special policy attention, national action and investment need be carried out in those 14 countries/territories.

New research demonstrated that 11 maternal infections per 1000 live births developed a severe outcome, but up to 15 women were affected in low- and middle-income countries [8]. Similarly, our study observed that the low and low-middle SDI countries/territories had the fastest downward trends of MSMI, but a disproportionately higher disease burden. Moreover, all ASR values and MMR were markedly negatively correlated with the corresponding SDI at the regional and national levels. Based on the definition of SDI and the above finding, we suggested that MSMI burden was associated with economic level, education level and awareness in health workers. Improvement in sanitation and access to routine prophylactic antibiotics during caesarean section have been shown to be effective and cost-effective strategies to reduce maternal death [31]. Unfortunately, approximately 10% of antibiotics were substandard and falsified in low- and middle-income countries, which could be a contributing factor to the high maternal mortality rate in these regions [32]. Low-quality medicines could increase mortality and morbidity and lead to additional costs [33–35]. Their potential negative health impact raised serious concerns. More efforts should be made to improve the quality of medicine. The production and sale of bogus or substandard drugs should be severely punished. The quality and safety of medicines need to be monitored effectively and continuously by government agencies in low- and middle-income areas.

Although the prevalence of sepsis-related maternal mortality was much higher in low and middle-income countries, sepsis was still one of the most important causes of maternal death in the developed world [9]. Therefore, a reduction in mortality caused by MSMI will need not only more investment or resources in public health but also a focus on more tailored prevention strategies and more effective clinical treatment, such as innovation in early diagnosis, point of care tests and targeted management. Undoubtedly, more clinical trial evidence is need to establish obstetrically appropriate infection or sepsis identification criteria.

The incidence of maternal sepsis shows geographic distribution differences around the world, with a high incidence occurring in Central and South Asia [11]. The death count also differed in various regions [20, 36]. In line with previous reports, the burden and trends of MSMI varied substantially in diverse regions and countries in our study. Central Sub-Saharan Africa and Eastern Sub-Saharan Africa were in the top 2 MSMI ASR and MMR. In the same manner, Somalia and the Central African Republic showed the highest ASDR, MMR and age-standardized DALY rate. Moreover, many countries still suffer from increases in ASDR and MMR, such as the Democratic Republic of the Congo, Zimbabwe, Central African Republic, Mauritius, Georgia, Kyrgyzstan, Kazakhstan and the United States. The heavy burden and upward trends of MSMI in some African and Asian countries may be attributed to low economic levels, poor medical care, lack of education and so on. Interestingly, the ASDR and MMR of MSMI were ascending in the United States with the highest economic levels, best medical environment and better education. Previous research showed a 10% per year increase in severe maternal sepsis and sepsis-related death in the United States between 1998 and 2008 [37]. The authors found that older than 35 years, smoking, no insurance and chronic comorbid conditions (such as diabetes mellitus) were common risk factors for pregnancy-associated sepsis [37]. Therefore, these high-risk women need closer monitoring and further studies focused on high-risk women are crucial steps in decreasing the mortality rate of maternal sepsis.

Late childbirth’ become an increasingly popular trend in many countries and regions [38, 39]. Currently, advanced maternal age is generally defined as pregnancy in women aged 35 years or older [40]. Late pregnancy was associated with a number of infectious diseases due to changes in immune responses [41]. Moreover, advanced maternal age was a risk factor for adverse maternal events, which has been widely acknowledged [38, 42–44]. In this context, a comprehensive report presenting the epidemiological burden and trends of MSIMI in women of different age is invaluable for policymakers to allocate healthy resources and Initiatives. In our study, we demonstrated that the burden of MSMI in younger women clearly exhibited a downward trend. However, mortality and DALY number in advanced maternal age did not show a decline until the early few years of the twenty-first century. The incident cases were increased slightly in older women, and the largest MMR was observed in the above 49 year group, followed by the 45–49 and 40–44 year group. This implied that older age partly accounted for a higher MMR, which was consistent with Kendle et al.’s finding that older women had the highest odds of sepsis-related in-hospital mortality [45]. We should not advocate late pregnancy and encourage women to give birth at an appropriate age.

Several limitations should also be noted when interpreting our results. Firstly, the availability and quality of primary data is major restriction of GBD assessment on the burden of diseases. Although amendments to data processing and complicated modelling can improve the accuracy of estimates when data are not available [10]. Further efforts are need to improve the availability and quality of data related to maternal mortality. Secondly, the burden of MSMI could be larger than estimated ones due to the difficulty in diagnosis of maternal sepsis. Given the above restrictions, our results should be interpreted carefully and cautiously.

## Conclusion

All in all, our results suggested that burden of MSMI were gradually declined in most countries and regions from 1990 to 2019, reflecting continued progress towards maternal health. But a few countries and regions still exhibited upward trends in ASR and MMR of MSMI. Hence, it posed challenge on reaching the WHO goal [28]. These analyses should inform the scientific priority of health policies, programmes, and funding to reduce maternal deaths at country and regional level.

## Supplementary Information

**Additional file 1. **The overview of the GBD 2019 database and the estimation methods for disease burden in MSMI.

**Additional file 2. **The SDI of different countries and territories in 2019.

**Additional file 3: Table S1.** The number of MSMI incidence, death, DALY globally from 1990 and 2019

**Additional file 4: Table S2.** The ASIR, ASDR and age-standardized DALY rate globally from 1990 and 2019

**Additional file 5: Table S3.** The number and rate of MSMI incidence, death, DALY in different age groups worldwide from 1990 to 2019

**Additional file 6: Table S4.** The number and age-standardized rate of MSMI incidence, death, DALY in different SDI from 1990 to 2019

**Additional file 7: Table S5.** The number of MSMI incidence, death, DALY in 21 different region in 1990 and 2019

**Additional file 8: Table S6.** The ASIR, ASDR and age-standardized DALY rate in 204 countries and territories in 2019

**Additional file 9: Table S7.** The EAPC of ASIR, ASDR, age-standardized DALY rate and MMR in 204 countries and territories in the last three decades

**Additional file 10: Table S8.** The MMR of MSMI in different age groups, 5 SDI quintiles, 21 region, and in 204 countries.

**Additional file 11: Figure S1.** The correlation between EAPC and ASIR in 1990 or SDI in 2019 in 204 countries and territories.

## Data Availability

The datasets used in the present study are available in GBD 2019. This data can be found here: http://ghdx.healthdata.org/gbd-results-tool.

## Abbreviations

MSMI: Maternal sepsis and other maternal infections
DALY: Disability-adjusted life year
MMR: Maternal mortality ratio
ASR: Age-standardized rate
EAPC: The estimated annual percentage change
SDI: Sociodemographic index
GBD: Global Burden of Disease database
