# Supplementary material for: The global burden and trends of maternal sepsis and other maternal infections in 204 countries and territories from 1990 to 2019
Source: BMC Infect Dis. 2021 Oct 18;21:1074. doi: 10.1186/s12879-021-06779-0 (PMC8524924; doi:10.1186/s12879-021-06779-0)
Supplement: Supplementary file 1 — Additional file 1. The overview of the GBD 2019 database and the estimation methods for disease burden in MSMI. [file 12879_2021_6779_MOESM1_ESM.docx]

**GBD Overview**
The Global Burden of Diseases, Injuries, and Risk Factors Study (GBD) provides a systematic scientific assessment of published, publicly available, and contributed data on global descriptive epidemiology. It estimates incidence, prevalence, mortality, years of life lost (YLLs), years lived with disability (YLDs), and disability-adjusted life-years (DALYs) due to 369 diseases and injuries, for two sexes, and for 204 countries and territories in the past decades. The numbers and rates of incidence, prevalence, years lived with disability (YLDs), and disability-adjusted life-years (DALYs) were estimated for the years 1990–2019, while deaths and years of life lost (YLLs) were estimated for 1980–2019. The cause and sequelae list were based on input from the Scientific Council and GBD collaborator network.

GBD Input data were extracted from censuses, household surveys, civil registration and vital statistics, disease registries, health service use, air pollution monitors, satellite imaging, disease notifications, and other sources. Cause-specific death rates and cause fractions were calculated using the Cause of Death Ensemble model and spatiotemporal Gaussian process regression. Cause-specific deaths were adjusted to match the total all-cause deaths calculated as part of the GBD population, fertility, and mortality estimates. Deaths were multiplied by standard life expectancy at each age to calculate YLLs. A Bayesian meta-regression modelling tool, DisMod-MR 2.1, was used to ensure consistency between incidence, prevalence, remission, excess mortality, and cause-specific mortality for most causes. Prevalence estimates were multiplied by disability weights for mutually exclusive sequelae of diseases and injuries to calculate YLDs. We considered results in the context of the Socio-demographic Index (SDI), a composite indicator of income per capita, years of schooling, and fertility rate in females younger than 25 years. Uncertainty intervals (UIs) were generated for every metric using the 25th and 975th ordered 1000 draw values of the posterior distribution.
**Definition of indicator**The GBD cause list is organized hierarchically. Levels 1 and 2 aggregate causes into general groupings. There are four cause groups at level 1: Communicable, maternal, neonatal, and nutritional diseases. The “maternal disorders” group which includes disability due to maternal causes, is at Level 3 under level 2 of “Maternal and neonatal disorders.” Level 4 includes 7 groups including 1) Abortion and miscarriage; 2) Ectopic pregnancy; 3) Obstructed labour and uterine rupture;4) Maternal haemorrhage; 5) Maternal sepsis and other maternal infections; 6) Maternal hypertensive disorders; and 7) Other [direct] maternal disorders. Indirect maternal disorders, late maternal deaths, and maternal deaths aggravated by HIV/AIDS did not have any estimated disability based on the premise that it is captured in the respective underlying causes.

Maternal sepsis and other maternal infections：

a. Maternal sepsis is defined as a temperature <36°C or >38°C and clinical signs of shock including systolic blood pressure <90 mmHg and tachycardia >120 bpm;

b. Other maternal infections are defined as any maternal infections excluding HIV, sexually transmitted infections, or are not believed to have epidemiologic relationship with pregnancy. Examples include urinary tract infections, mastitis, candidiasis, and bacterial vaginosis during pregnancy.

**Input data**

Systematic literature reviews have been completed on May 10, 2019. In addition, GBD searched ministry of health websites for pregnancy complication data and used Confidential Enquiry and other sources used in our maternal mortality analyses when they presented data on pregnancy complications. GBD also performed snowball searches for abortion reporting and surveillance data systems, finding multiple such systems throughout high-income countries and several geographies in Central and Eastern Europe. Inpatient and outpatient data were used, as were claims data from Taiwan and Singapore as well as MarketScan in the United States.All data were either extracted as incidence ratio (number of events / live birth) or, if data were only available with population as the denominator, they were converted to incidence ratio using GBD 2019 age-specific fertility rate (number of live births / population). All data were extracted in standard fashion, and were uploaded and stored on a centralised SQL database.

In locations with low-quality, or no vital registration, maternal mortality metrics can be found in surveillance, surveys, census, and sibling history data sources. The best data have death counts due to maternal causes and the total number of deaths for women within the reproductive ages of 10–54 by year. If a data source is missing these components, creating a complete cause list is necessary by using live births and all-cause mortality deaths. Though death counts are the preferred metric, maternal mortality is often measured by using the maternal mortality ratio (MMR), which is easily converted to deaths by using live births. The China Maternal and Child Surveillance data is adjusted by scaling data from the strata to the province level.

**Data processing**

The first step of data processing was age-sex splitting. For any datum that did not entirely fit within a GBD age group or was for both sexes combined, the observation was split to be multiple age specific and sex-specific data points based on the age and sex pattern predicted by GBD 2017 DisModMR 2.1 models.

The second step was crosswalking all data from alternate to reference definitions. For all other models, we adjusted data to the reference category for each cause by age using Meta-Regression-Bayesian, Regularized, Trimmed (MR-BRT), a meta-analytic tool developed for GBD 2019. In accordance with GBD 2019 principles for data processing, to make data comparable, staffs of GBD began by evaluating the number of observations of each alternate definition that matched with a corresponding observation from the reference definition. They excluded some alternative definitions from this process, e.g. studies reporting chronic hypertension and studies reporting severe diagnoses of maternal disorders except for sepsis and eclampsia. The standard error of the ratio was calculated using the delta method. The details of each of the crosswalks are described below. All data sources that only reported event rates for severe maternal morbidity or “near miss” were excluded as a reliable crosswalk model could not be developed.

More specifically, puerperal sepsis cases reported in literature studies were the reference category. We crosswalked claims data to inpatient data by age. After this adjustment we crosswalked all of the clinical data to the literature data by age. The age pattern for the claims to inpatient crosswalk was significant with an increase with age until age 40. The age pattern of clinical to literature was slightly decreasing with age. Inpatient hospital data were the reference for other maternal infections. We crosswalked claims data to inpatient hospital data by age. The age pattern shows a steep increase in the ratio from ages 10 to 35.

**Modelling strategy**

Incidence ratio for each age-sex-location-year in the GBD 2019 location hierarchy were estimated using DisMod-MR 2.1. A series of country covariates were chosen to help drive the magnitude of estimates in areas of sparse or absent data. GBD staffs included the respective log transformed maternal mortality ratio (MMR) for each maternal disorder that was estimated as a country level covariate for almost every model. Puerperal sepsis used the log transformed age standardized death rate (LN-ASDR) as a covariate, instead of MMR. No specific age or slope priors were used. All models were run with a time window of five years. **Incidence estimation**

All age-specific ratios were then converted to incidence rates by multiplying by live births per population. Maternal sepsis was assigned a duration of five days (+/-2) and, based on the same data identified in our review of pelvic inflammatory disease, 9% (7.7% - 10%) of incident cases of puerperal sepsis were estimated to continue on to have secondary infertility due to maternal sepsis. GBD staffs apply this proportion to the incidence results of puerperal sepsis and use them as input data for a full compartment DisMod-MR 2.1 model. Other maternal infections were assigned a wide potential duration of 15 to 45 days (mean 30).
**Uncertainty and model selection**For all maternal disorders, uncertainty bounds include uncertainty due to input data, crosswalks from non-reference definitions, uncertainty in numerical solutions (posteriors) of each DisMod-MR 2.1 model. In consultation with GBD researchers and collaborators, final models were selected on a combination of qualitative and quantitative goodness of fit to input data, plausibility of geographic and temporal trends, consistency of age pattern, and, when available, comparison with other published studies on the epidemiology of pregnancy complications. Directionality, magnitude, and plausibility of study-level and country-level covariates were also considered in the process of model development. Of note, due to the nature of statistical modelling, final results do not always cover the values reported in input data.

**Data analysis**ASRs were calculated on the basis of the following formula:

$ASR=\frac{\sum_{i=1}^{A} a_{i}w_{i}}{\sum_{i=1}^{A} w_{i}}$×100,000

The ASR (per 100,000 population) is equal to the sum of the product of the specific age ratio (ai) in age group i and the number (or weight) (wi) of the selected reference standard population group i divided by the sum of number (or weight) of the standard population, i.e.

Meanwhile, EAPC, which is approximately equal to the annual change for a specified range, was calculated using the following regression model to assess the trends in ASR: Y=α+βX+ε, where y refers to ln(ASR), x represents calendar year, ε means error term, and β determines the positive or negative trends in ASR. The EAPC could be given by 100*(exp(β)-1), as well as its 95% confidence interval (CI).

Disability adjusted life-years were also defined as years of healthy life lost, estimated by the sum of the YLLs and the YLDs. YLLs, the years of life lost due to premature mortality, are the multiplication of deaths and a standard life expectancy at each age of death. In each population greater than 5 million, the standard life expectancy was estimated from a life table which includes the lowest observed mortality at each age group. YLDs were defined as the years lived with any short-term or long-term health loss weighted for severity by the disability weights, calculated by multiplying prevalence estimate and the disability weight of each mutually exclusive sequela for prostate cancer, such as disability due to incontinence and impotence from prostatectomy.
