# Supplementary material for: The global burden and trends of maternal sepsis and other maternal infections in 204 countries and territories from 1990 to 2019
Source: BMC Infect Dis. 2021 Oct 18;21:1074. doi: 10.1186/s12879-021-06779-0 (PMC8524924; doi:10.1186/s12879-021-06779-0)
Supplement: Supplementary file 11 — Additional file 11: Figure S1. The correlation between EAPC and ASIR in 1990 or SDI in 2019 in 204 countries and territories. [file 12879_2021_6779_MOESM11_ESM.docx]

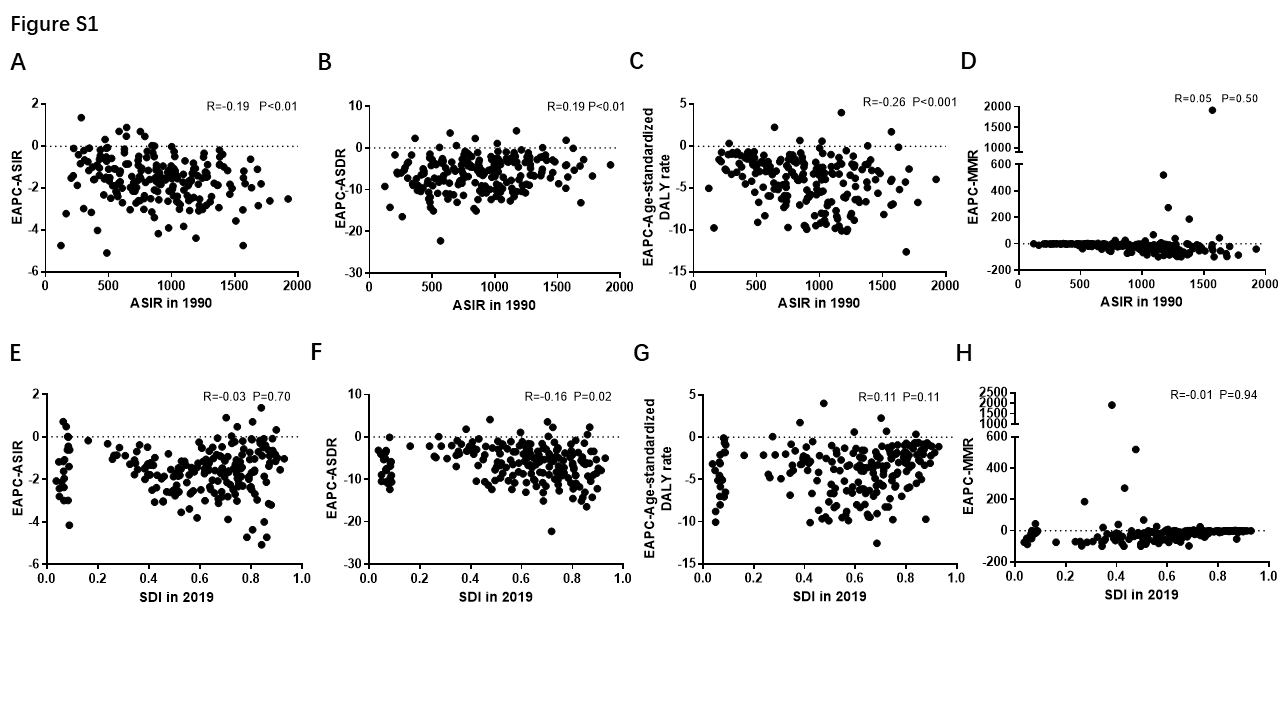


Figure S1. The correlation between EAPC and ASIR in 1990 or SDI in 2019 in 204 countries and territories.

The ASIR in 1990 were negatively correlated with EPAC of ASIR **(A)**, ASDR **(B)** and age-standardized DALY rate **(C)**, but not with EPAC of MMR **(D)**. The SDI in 2019 were negatively correlated with EPAC of ASDR **(F),** but not with EPAC of ASIR **(E)**, age-standardized DALY rate**(G)** as well as MMR **(H)**.
